# Deep Learning-Based Multi-Cancer Analysis for Predicting Disease-Free Survival Across Multiple Cancer Types

**DOI:** 10.3390/cancers18182948

**Published:** 2026-09-11

**Authors:** Siteng Chen, Encheng Zhang, Fukang Sun, Feng Gao, Da Huang, Dawei Wang, Rong Na, Liren Jiang, Ning Zhang

**Affiliations:** 1 Department of Urology, Renji Hospital, Shanghai Jiao Tong University School of Medicine, Shanghai 200001, China; 2 Department of Urology, Shanghai General Hospital, Shanghai Jiao Tong University School of Medicine, Shanghai 200080, China; 3 Department of Urology, Ruijin Hospital, Shanghai Jiao Tong University School of Medicine, Shanghai 200025, China; 4 Pathology Center, Shanghai General Hospital, Shanghai Jiao Tong University School of Medicine, Shanghai 200080, China; 5 Division of Urology, Department of Surgery, LKS School of Medicine, The University of Hong Kong, Hong Kong, China; 6 Division of Urology, Department of Surgery, Queen Mary Hospital, Hong Kong, China

**Keywords:** deep learning, multiple cancer types, whole-slide image, prognosis, nomogram

## Abstract

**Background**: Artificial intelligence-derived parameters hold substantial promise as indicators for tumor prognosis prediction and treatment guidance. However, existing studies have not sufficiently addressed the application of these parameters across different cancer types. **Methods**: We employed a deep learning algorithm to conduct a multi-cancer analysis for disease-free survival (MC-DFS) prediction using 8856 cases with associated whole-slide images and clinical data. The training cohort consisted of 7392 cases from the TCGA set (24 cancer types), and the independent external validation cohort comprised 1464 cases from the CPTAC and General Hospital sets (9 cancer types). A nomogram prediction signature for disease-free survival (NOMO) was developed by integrating the MC-DFS, tumor stage, and patient age. The prognostic model’s performance was validated in an independent cohort. **Results**: In the training and validation cohorts, the MC-DFS model achieved area under the curve (AUC) values of 0.750 and 0.682, respectively. It effectively differentiated patients with poorer disease-free survival, with hazard ratios of 4.823 (95% CI: 4.343–5.356, *p* < 0.0001) in the training cohort and 2.092 (95% CI: 1.472–2.971, *p* < 0.0001) in the validation cohort. Each cancer subtype’s analysis confirmed the model’s robust performance. Additionally, using nomogram analysis, we developed a multi-model prediction signature for disease-free survival across multiple cancer types based on MC-DFS and the clinicopathologic features in the training cohort. This enhanced model offers more precise risk stratification for stage I malignancies and complements the existing tumor staging systems by identifying high-risk patients. **Conclusions**: The newly developed MC-DFS shows marked improvements in prognostic predictions across multiple cancer types. With further validations across multiple centers, this nomogram prediction system could become a valuable practical tool for managing various cancers.

## Simple Summary

Artificial intelligence-derived parameters hold promise for tumor prognosis, yet their application across cancer types remains underexplored. We developed a deep learning-based multi-cancer disease-free survival (MC-DFS) model using 8856 cases with whole-slide images and clinical data. The training cohort comprised 7392 cases from TCGA (24 cancer types), and independent external validation included 1464 cases from CPTAC and a hospital cohort (9 cancer types). Subsequently, a nomogram (NOMO) integrating MC-DFS, tumor stage, and age was constructed. In the training and validation cohorts, MC-DFS achieved AUCs of 0.750 and 0.682, respectively, with hazard ratios of 4.823 and 2.092 (both *p* < 0.0001). The model performed robustly across cancer subtypes, with the nomogram improving risk stratification, particularly for stage I malignancies, complementing existing staging. With multicenter validation, this system could become a practical tool for managing diverse cancers.

## 1. Introduction

The number of new cancer cases in the USA was projected to reach 2,001,140 in 2024 and is expected to continually rise each year. Fortunately, due to medical advancements over the past three decades, the current cancer death rate has significantly decreased [1]. These improvements are primarily due to rapid developments in cancer therapies, diagnosis, and prevention, which increasingly require precision and accuracy from clinicians [2]. Despite these advances, cancer remains the second leading cause of death in the USA and was expected to result in 611,720 fatalities in 2024 [1,3]. Thus, developing novel indicators for precision treatment is crucial.

Artificial intelligence (AI) analysis holds substantial promise for providing clinicians with ideal indicators to predict tumor prognosis and guide treatment. Machine learning of high-throughput omics data and advanced imaging techniques has proven to be clinically valuable [4,5]. Specifically, deep convolutional neural networks reliably diagnose and predict various malignancies, and when combined with digitized pathological slides, they have been used to estimate survival outcomes in cancer patients [6,7,8,9,10,11]. While analyzing individual factors yields only modest prognostic gains, integrating multimodal information into a single signature substantially improves the accuracy of cancer prognosis prediction [12]. Deep learning, a product of advanced AI techniques, employs multiple artificial neuron layers to mimic human cognitive processes and excels at complex data recognition and generation, thereby achieving high performance in diverse medical applications such as disease diagnosis and drug discovery [13,14,15,16]. With the rapid evolution of AI, clinicians will ultimately benefit from these innovations in delivering precise, individualized treatment [17]. Furthermore, emerging evidence supports the use of deep learning in forecasting patient responses to immune checkpoint inhibitors by integrating tumor microenvironment features and peripheral immune metrics, thereby expanding the therapeutic reach of predictive analytics [18]. Given the rapid, iterative evolution of computational intelligence, clinicians can ultimately harness these technological breakthroughs to operationalize precision medicine, delivering dynamically adaptive, individually calibrated therapeutic regimens in routine oncology workflows [19,20]. However, to date, most studies were not conducted with the aim of developing a generalized parameter applicable across different cancer types; this is a critical gap that limits the translation of AI-based tools into routine clinical practice.

Here, we introduce a deep learning-based multi-cancer analysis for disease-free survival across multiple cancer types, involving 8856 cases with whole-slide images (WSIs) and associated clinical information. Our integrated analysis demonstrates the model’s generalized performance across multiple cancer types, highlighting its high accuracy and potential for clinical application.

## 2. Methods

### 2.1. Patient Cohorts and Data Resource

In this study, we retrieved two multi-cancer cohorts from the Cancer Genome Atlas (TCGA), the Clinical Proteomic Tumor Analysis Consortium (CPTAC) through the Cancer Imaging Archive [21,22,23], and the General Hospital. Only a specific sample with a single malignant tumor diagnosis was included for analysis. In addition, included patients had complete clinical follow-up data and hematoxylin–eosin (H&E)-stained WSIs scanned at 20× objective magnification (spatial resolution: approximately 0.5 μm/pixel). Ethical approval of our study was obtained from the Human Ethics Committee of Shanghai General Hospital (Approval Letter No. 20240507040632232), which also granted a waiver of informed consent as this was a retrospective analysis.

The TCGA set was used as the training cohort, which contained 7392 WSIs from 24 tumor types, including adrenocortical cancer (ACC), bladder cancer (BLCA), breast cancer (BRCA), cervical cancer (CESC), cholangiocarcinoma (CHOL), colon adenocarcinoma (COAD), esophageal carcinoma (ESCA), head and neck cancer (HNSC), kidney chromophobe (KICH), kidney renal clear cell carcinoma (KIRC), kidney renal papillary cell carcinoma (KIRP), liver hepatocellular carcinoma (LIHC), lung adenocarcinoma (LUAD), lung squamous cell carcinoma (LUSC), mesothelioma (MESO), ovarian cancer (OV), pancreatic cancer (PAAD), prostate cancer (PRAD), rectum adenocarcinoma (READ), melanoma (SKCM), stomach adenocarcinoma (STAD), testicular germ cell tumor (TGCT), thyroid carcinoma (THCA), and endometrioid cancer (UCEC). Another 1464 WSIs based on 9 types of malignant tumors were also recruited from the CPTAC and General Hospital sets, including BLCA, BRCA, COAD, KIRC, KICH, KIRP, LUAD, PRAD, and UCEC, which were further applied for the independent external validation of the MC-DFS.

Patients who underwent radical surgery at Shanghai General Hospital between January 2008 and December 2021 were enrolled in this study, as well as patients from the TCGA and CPTAC cohorts with available H&E-stained WSIs at 200× magnification and immune subtype data. All patients met the following inclusion criteria: (i) complete follow-up data for disease-free survival (DFS); (ii) availability of paraffin-embedded tumor tissue and corresponding pathological information; and (iii) no history of other malignancies.

### 2.2. Process of Formalin-Fixed Tumor Slide Through Hematoxylin–Eosin

In the General Hospital cohort, the formalin-fixed tumor samples were sectioned to create histologic sections (5 μm), which were subsequently stained with hematoxylin (Sigma-Aldrich, St. Louis, MO, USA) for 5 min and differentiated with acid ethanol (Sigma-Aldrich) at a 1% concentration. After being rinsed in distilled water, the section was further stained with eosin (Sigma-Aldrich) for 3 min, dehydrated through graded alcohol, and cleared in xylene (Sigma-Aldrich). Finally, H&E-stained slides were scanned and collected through a Leica DM2500 LED fluorescence microscope (Leica Microsystems, Wetzlar, Hesse, Germany) under 200 times magnification.

### 2.3. Image Processing and Feature Extraction

We applied a clustering-constrained attention multiple instance learning method to accurately identify the sub-regions of the WSIs [5]. Macenko normalization was used to standardize H&E stain color to a reference template, and random color augmentation was applied during training to improve generalization. The attention-based multiple instance learning framework was chosen because it provided instance-level interpretability (attention scores for each patch), enabling visualization of histological features driving predictions. The attention module consists of two fully connected layers: the learning rate was set to 1 × 10^−4^ with the Adam optimizer, and early stopping with validation loss patience was set to 20 epochs. Model training was performed on an NVIDIA A100 GPU (40 GB memory) with 64 CPU cores, which required approximately 72 h for 60 epochs. The original WSIs were first segmented to remove non-tissue regions. The reserved tissue regions of each slide were further segmented into many smaller patches, which could act as direct inputs to convolutional neural networks. The patches were extracted at 256 × 256 pixels. We applied an instance-level clustering method to convert tissue patches into low-dimensional feature embeddings to learn and extract the class-specific features. A 2048-dimensional feature vector was acquired from each cropped patch, which was further used for model training and hyperparameter optimization through a weak supervision training strategy (MC-DFS version 2024, code available on GitHub at https://github.com/mahmoodlab/clam, accessed on 1 July 2024).

### 2.4. Model Training and Hyperparameter Optimization

During model training, WSIs were randomly sampled and participated in the training with a batch size of one. The sampling probability of each WSI was inversely proportional to the frequency of the ground-truth class. Weights and bias parameters of the attention module were initialized randomly and trained end-to-end with the rest of the model as previously reported [5]. We used the outputs of the attention network to generate pseudo labels to supervise the clustering in each iteration. To mitigate overfitting, we implemented multiple regularization strategies throughout the training pipeline. First, dropout layers (rate = 0.25) were incorporated within the two fully connected layers of the attention module, providing stochastic regularization during training. Second, weight decay was applied to all trainable parameters during optimization with the Adam optimizer. Third, random color augmentation was applied to input patches during training to enhance generalization by increasing the diversity of the training data. Fourth, early stopping was employed with a validation loss patience of 20 epochs, and the model achieving the lowest validation loss was automatically saved and tested on the test set. The external validation cohorts were strictly held out and were never used for any hyperparameter tuning or model selection decisions. The deep learning-based model was trained for at least 60 epochs. The total loss of the slide was set as the sum of both the instance-level clustering loss and the slide-level classification loss with optional scaling. We implemented a weighted sampling strategy during training, where the sampling probability of each WSI was inversely proportional to the frequency of the ground-truth class. The model with the lowest validation loss was automatically saved and tested on the test set. The cut-off value of each prediction model was determined using Youden’s Index, which maximizes the sum of sensitivity and specificity (Youden’s J = sensitivity + specificity − 1).

### 2.5. Nomogram Prediction for Disease-Free Survival in the Multi-Cancer Cohort

**Figure 1:**
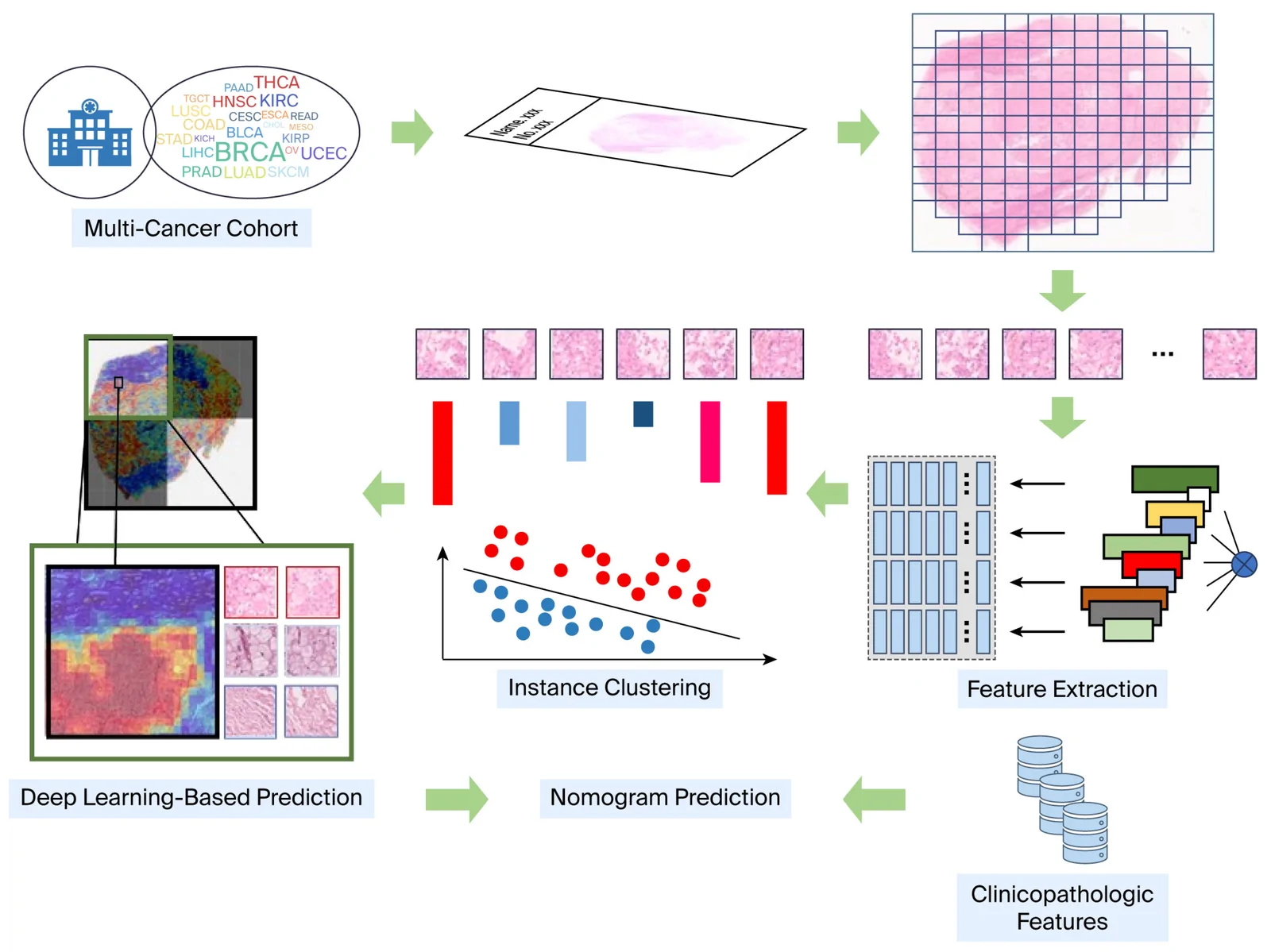
An analysis pipeline of this study.

Through nomogram analysis, we developed a multi-model prediction signature for disease-free survival across multiple cancer types based on our MC-DFS and the clinicopathologic features in the training cohort. We further validated the multi-model signature in the independent validation cohort. A detailed outline of this study is shown in Figure 1.

### 2.6. Statistical Analysis

In this study, we applied Python 4.0 and R 3.6.2 for model training and statistical analyses. Kaplan–Meier (KM) survival analysis with a hazard ratio (HR) and 95% confidence interval (CI) was carried out through a log-rank test to compare different survival outcomes, and disease-free survival was defined as the time from primary surgery to first tumor progression, recurrence, or last follow-up for censored patients. The area under the curve (AUC) value obtained from the receiver operating characteristic (ROC) curve was also used to evaluate the specificity and sensitivity of the prediction model, and a *p*-value less than 0.05 was set as the significance level. A comparison of AUCs among different groups was carried out using the DeLong method.

## 3. Results

### 3.1. Clinical and Pathological Characteristics

The clinical and pathological features of the training (*n* = 7392) and verification (*n* = 1464) cohorts are shown in Table 1, with mean follow-up durations of 36.01 ± 35.45 and 40.56 ± 25.91 months, respectively. BRCA was the largest proportion in the training cohort (13.7%), while KIRC was the largest in the verification cohort (24.8%). The primary clinical information of the included samples is shown in Table 1.

### 3.2. Performance of the MC-DFS Across Multiple Cancer Types

**Figure 2:**
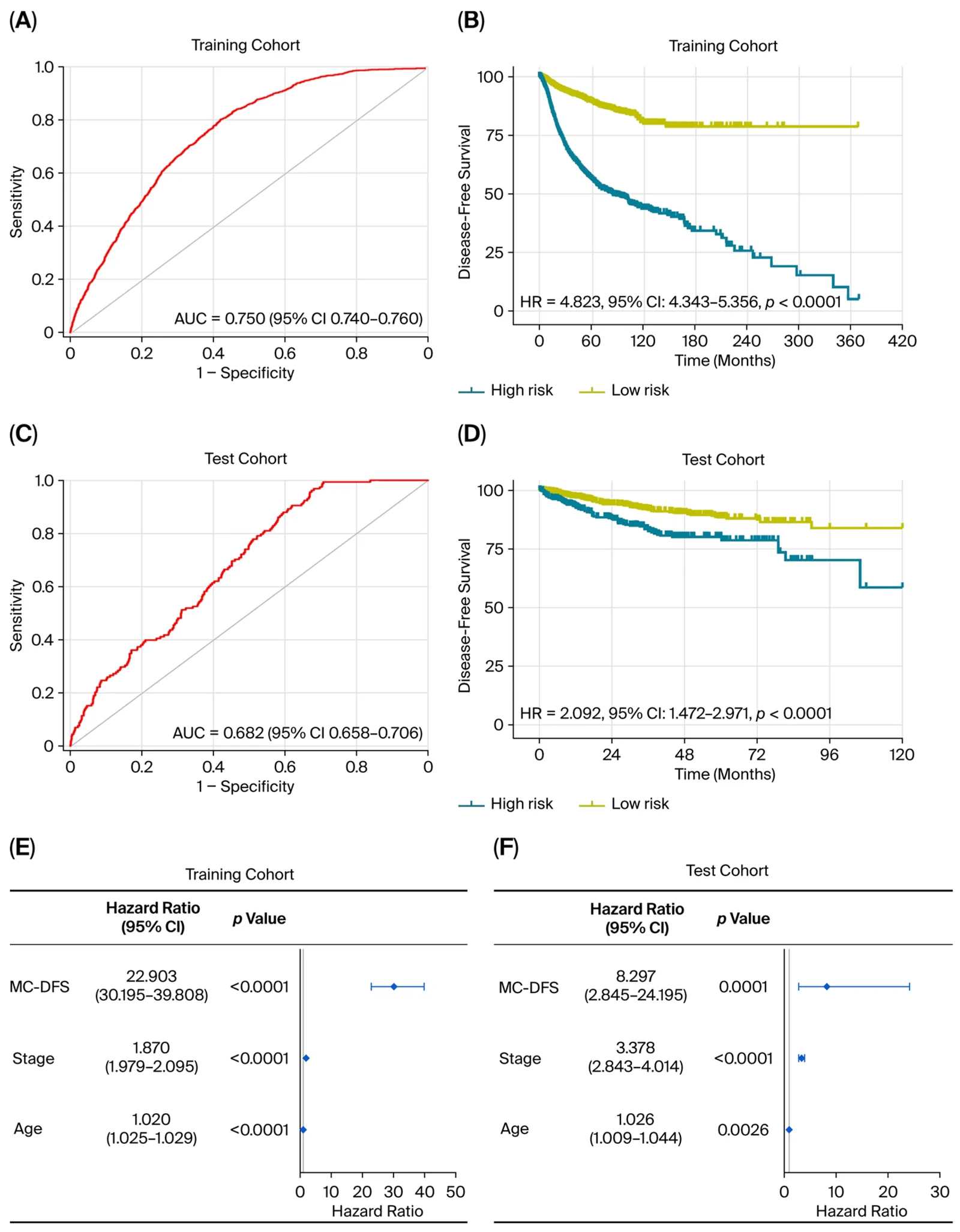
Performance of the multi-cancer prediction signature for disease-free survival (MC-DFS). (**A**,**C**) Receiver operating characteristic analysis for MC-DFS in the training and verification cohorts. (**B**,**D**) Kaplan–Meier survival analysis of disease-free survival predicted by MC-DFS in the training and verification cohorts. (**E**,**F**) Cox regression analyses indicated that MC-DFS could act as an independent risk factor in the training and verification cohorts.

Based on our deep learning-based pathological strategy, we developed the MC-DFS across multiple cancer types with the lowest internal validation loss. ROC analysis revealed that our MC-DFS achieved promising prediction accuracy in the training cohort, with an AUC of 0.750 (0.749–0.760, *p* < 0.0001, Figure 2A). When the cut-off value of MC-DFS was set as “0.3117”, the model achieved the best performance in predicting DFS. Patients with MC-DFS higher than “0.3117” were grouped as the MC-DFS_high-risk_ group and as the MC-DFS_low-risk_ group otherwise. Further KM survival analysis revealed that our MC-DFS could distinguish patients with worse DFS in the multi-cancer training cohort, with an HR of 4.823 (95% CI: 4.343–5.356, *p* < 0.0001, Figure 2B). An attention heatmap for MC-DFS interpretability indicated that the model primarily focuses on tumor areas with high nuclear pleomorphism. The high-attention regions (red areas in heatmaps) consistently corresponded to tumor areas exhibiting high nuclear pleomorphism, including marked variation in nuclear size and shape, irregular nuclear contours, and prominent nucleoli. Low-attention areas included stromal regions, necrotic areas, and lymphocyte aggregates, confirming the model’s biological relevance rather than artifact-driven attention.

Verification of the MC-DFS was also performed in an independent multi-cancer patient cohort. The AUC value for the CPTAC set was 0.701 (0.660–0.739), and that for the General Hospital cohort was 0.687 (0.656–0.717). As shown in Figure 2C, our MC-DFS achieved an AUC of 0.682 (0.65–0.706) in predicting the DFS of patients in the independent verification cohort. With the same cut-off value in the training cohort, patients with MC-DFS_high-risk_ were also associated with worse DFS, with an HR of 2.092 (95% CI: 1.472–2.971, *p* < 0.0001, Figure 2D) in the independent verification cohort, which revealed the generalization and replicability of our deep learning-based prediction signature. Further subgroup analyses based on cancer types indicated that our MC-DFS could achieve a high AUC value (more than 0.65) in 66.7% (16/24) of all tumor types (Table 2). Cox analysis revealed that our MC-DFS could act as an independent risk factor in 79.2% (19/24) of all tumor types in the training cohort and 77.8% (7/9) in the verification cohort, respectively (Table 3) (Figure 2E,F).

### 3.3. Construction of a Nomogram Prediction for Disease-Free Survival in the Multi-Cancer Cohort

**Figure 3:**
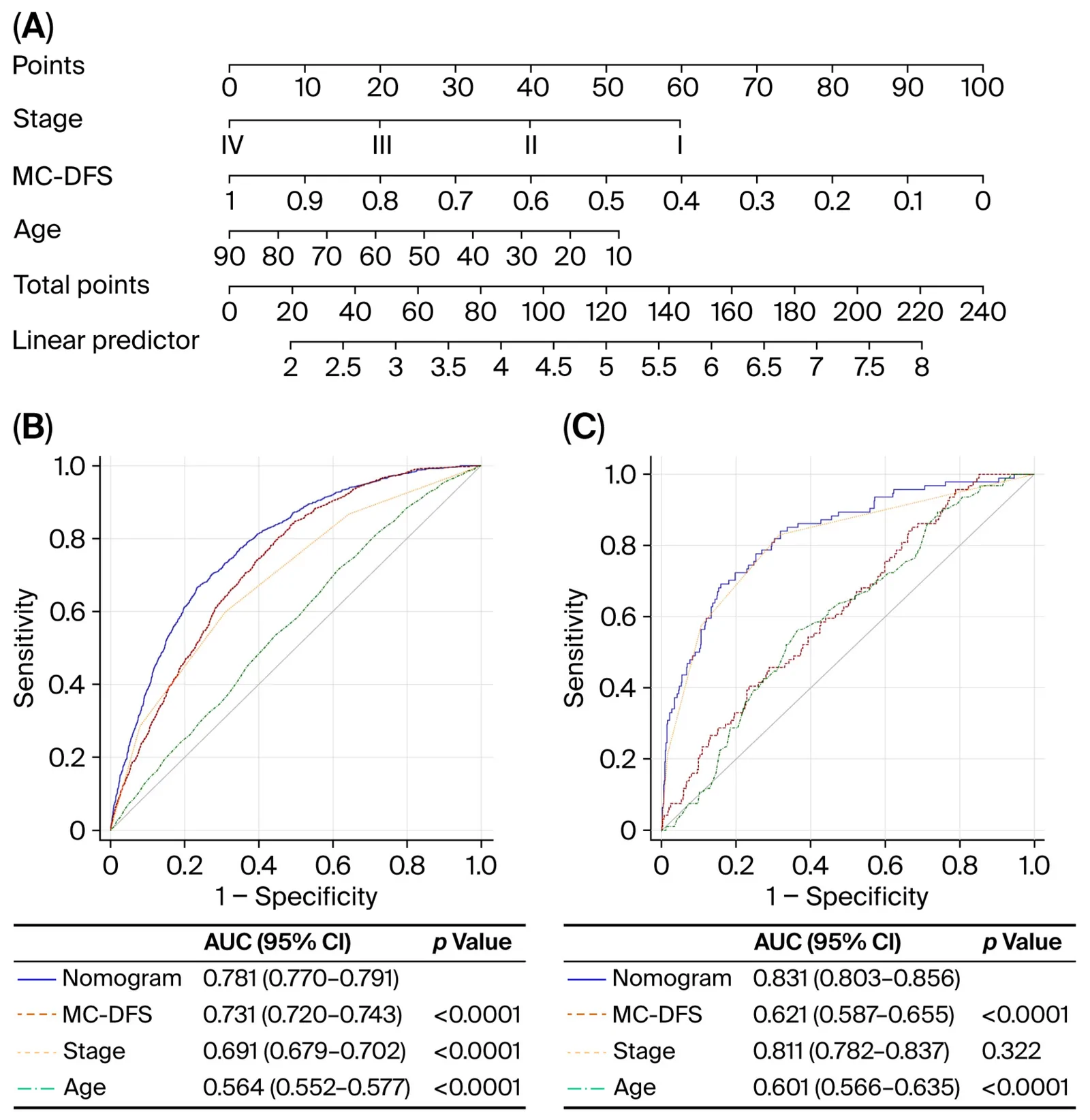
Construction of a nomogram prediction for disease-free survival in the multi-cancer cohort. (**A**) The nomogram prediction (NOMO) for disease-free survival in the training cohort. (**B**) Receiver operating characteristic analysis for NOMO in the training cohort. (**C**) Receiver operating characteristic analysis for NOMO in the verification cohort.

**Figure 4:**
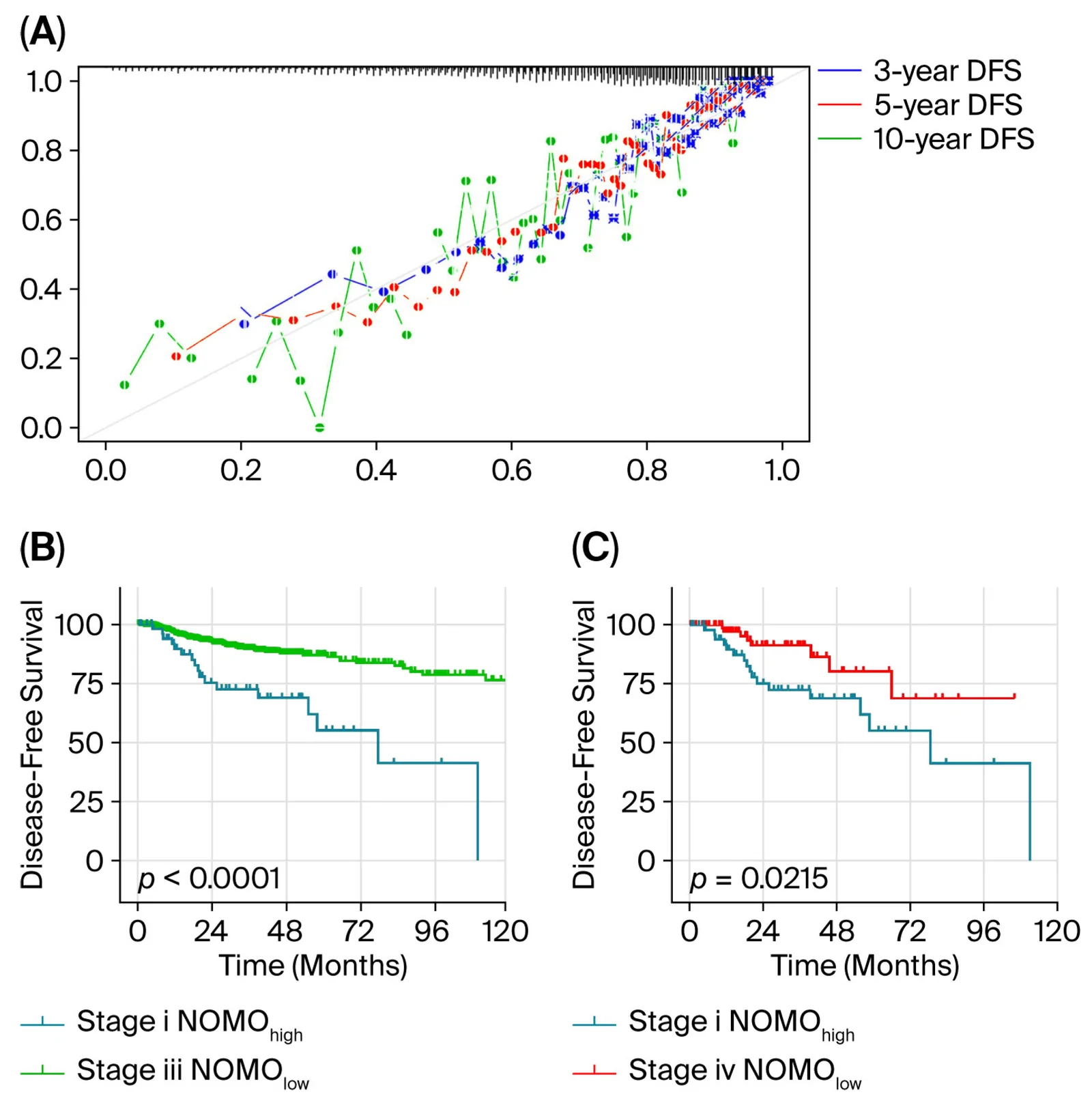
NOMO could provide complements to the current tumor staging system across multiple cancer types. (**A**) Calibration plots confirmed the accuracy of NOMO in predicting 3-year (blue), 5-year (red), and 10-year (green) DFS in the multi-cancer cohort. Kaplan–Meier survival analysis for disease-free survival between patients with (**B**) stage I NOMO_high_ and stage III NOMO_low_ and (**C**) stage I NOMO_high_ and stage IV NOMO_low_.

We developed a nomogram prediction (NOMO) for disease-free survival in the training cohort by combining MC-DFS, tumor stage, and patient age (Figure 3A). Through ROC analysis, we found that NOMO achieved better performance in predicting DFS status with an AUC of 0.781 (0.770–0.791, Figure 3B), which was significantly higher than that for MC-DFS (AUC = 0.731, *p* < 0.0001), tumor stage (AUC = 0.691, *p* < 0.0001), and patient age (AUC = 0.564, *p* < 0.0001) in the training cohort excluding patients without stage or age information, with a C-index of 0.750. Similar results were also verified in the independent verification cohort (Figure 3C). Calibration plots also confirmed the accuracy of NOMO in predicting 3-year, 5-year, and 10-year DFS in the multi-cancer cohort (Figure 4A).

With the best cut-off value (103.67) through ROC analysis in the training cohort, patients with NOMO scores higher than 103.67 were classified as NOMO_high-risk_, a group that was significantly associated with worse DFS, with an HR of 7.874 (95% CI: 6.918–8.962, *p* < 0.0001). Similar results were also verified in the independent verification cohort, with an HR of 9.996 (95% CI: 4.569–21.870, *p* < 0.0001).

### 3.4. NOMO Could Provide Complements to the Current Tumor Staging System Across Multiple Cancer Types

Stage I malignant tumors were expected to be associated with better clinical outcomes. However, when we performed further risk stratification through MC-DFS in patients with stage I tumors, we found that about 3% of these patients might suffer from a worse prognosis. As shown in Figure 4B, the DFS of patients with stage I but NOMO_high-risk_ was significantly shorter compared with that of patients with stage III but MC-DFS_low-risk_ (HR = 3.479, 95% CI: 1.489–8.126, *p* < 0.0001). Similar results were also observed in patients with stage I but NOMO_high-risk_ compared with those of patients with stage IV but NOMO_low-risk_ (HR = 2.517, 95% CI: 1.146–5.528, *p* = 0.0215; Figure 4C). These results suggest that our NOMO could provide more accurate risk stratification for stage I malignant tumors and complement the current tumor staging system to avoid overlooking high-risk patients.

## 4. Discussion

AI has profoundly influenced cancer diagnosis and treatment decision-making for pathologists and clinicians [24,25,26,27]. The integration of machine learning algorithms with clinical imaging has fostered advancements in high-throughput omics [28], achieving high accuracy in both diagnosis and disease outcome prediction. Radiomics, leveraging radiological images, offers a non-invasive approach that benefits patients. However, it may miss crucial histological features in tumor cells and the extracellular matrix due to the inherent limitations of radiological images.

Pathomics, which combines machine learning with digitized pathology, holds potential for personalized treatment. It surpasses radiomics by automatically extracting and analyzing pathological features from WSIs, providing more detailed and precise image-based features for accurate analyses [29,30]. Current methods such as topological analysis, morphological assessments, and convolutional neural networks explore complex pathological features across various magnifications, capturing both tissue and cellular structures of tumors [31]. WSIs also facilitate remote and advanced AI-driven pathological diagnoses [32]. Although research on WSI-based pathomics for different cancer types is progressing, most studies still focus on a single cancer type [33]. With the advent of deep convolutional neural networks, a generalized analysis of intra- and inter-tumor heterogeneity at the histological level is now feasible across multiple cancers.

The TNM staging system remains the primary clinical indicator for treatment decisions based on tumor severity [34]. Nevertheless, it can overlook the histological variations among patients within the same TNM stage, failing to account for features like polytypic nuclei and high nucleoplasm ratios, which typically indicate poor differentiation and unfavorable outcomes [34,35]. Our model, analyzed by an automated deep learning algorithm without pathologist annotations, minimizes biases from varying diagnostic criteria and captures subtle features such as the spatial organization of cancer cells, their stromal interactions, and adjacent immune cells, contributing to survival risk assessments. These common histological features, discernible across different cancers, enhance our deep learning-based multi-cancer analysis [34].

In this study, we applied a multi-cancer prognosis prediction analysis using 8856 WSIs and associated clinical data. The MC-DFS demonstrated prediction accuracies with AUCs of 0.750 and 0.682 in the training and verification cohorts, respectively. The integration of MC-DFS with clinical data significantly enhanced performance, illustrating the NOMO model’s potential to complement existing risk stratifications and improve clinical applications across multiple cancer types.

In routine clinical practice, prognosis assessment for cancer patients relies primarily on TNM stage, pathological differentiation, and individual clinician experience—an approach that is often subjective and may lead to suboptimal treatment planning and unfavorable outcomes. Furthermore, histological patterns vary not only across different cancer types but also within the same type depending on the degree of differentiation, which introduces substantial difficulty and inaccuracy in pan-cancer prognosis prediction. In contrast, the proposed nomogram offers a more visual and concise tool that integrates multimodal information, allowing clinicians to evaluate potential outcomes with greater clarity. Despite using a more feasible and cost-effective method, our nomogram achieves AUC values comparable to those of significantly more expensive models that incorporate, at the very least, genomic sequencing data, underscoring its strong potential for future clinical translation [34,36,37,38,39]. Consequently, our nomogram strikes a pragmatic balance between affordability and predictive power—a critical advantage over sequencing-dependent alternatives that remain inaccessible in many resource-limited settings. This nomogram might bridge the gap between affordability and predictive performance. It addresses a major barrier to the widespread adoption of AI-based tools: the high cost of multi-omics integration. As a result, it promises to democratize data-driven, individualized cancer care across diverse resource settings.

Compared with routine clinical practice, MC-DFS enables a more intuitive and concise assessment of the possible outcomes for cancer patients based on integrated information. Currently, clinicians primarily rely on TNM stage, pathological differentiation, and personal experience to evaluate prognosis, which may lead to suboptimal treatment planning and unfavorable clinical outcomes. Moreover, histological patterns vary not only across different cancer types but also within the same cancer type depending on the degree of differentiation. This heterogeneity introduces difficulty and compromises the accuracy of multi-cancer prognosis prediction. Our MC-DFS adopts a more practical and cost-effective approach, achieving AUC values comparable to those of more expensive models that incorporate, at the very least, genomic sequencing data (AUC range: 0.530–0.790), thereby highlighting its potential for future clinical applications [34,36,37,38,39].

The NOMO developed in this study offers several practical advantages that may facilitate its translation into clinical practice. First, unlike multi-omics-based prognostic models that require genomic sequencing, proteomic profiling, or transcriptomic data—which remain costly and inaccessible in many healthcare settings—our approach relies solely on routinely acquired H&E-stained slides and basic clinical information (age and tumor stage). This makes NOMO a cost-effective and widely accessible tool that could be deployed even in resource-limited environments where advanced molecular testing is unavailable. We regard the NOMO as a proof-of-concept tool that demonstrates the feasibility and promise of deep learning-based cancer prognosis prediction, rather than a mature clinical instrument ready for immediate deployment.

Beyond demonstrating the feasibility of a pan-cancer pathomics model, our findings highlight a crucial step toward democratizing precision oncology. Most existing prognostic tools that achieve comparable accuracy rely on multi-omics integration, including genomic or transcriptomic sequencing, which imposes substantial financial and infrastructural burdens. In contrast, our MC-DFS model extracts prognostic information directly from routine H&E-stained whole-slide images, a modality already generated during standard diagnostic workup. By forgoing additional sequencing, the nomogram maintains high discriminatory performance while remaining accessible to resource-limited settings. Furthermore, because the model learns from histomorphological patterns shared across cancer types, such as tumor cell spatial organization, stromal architecture, and immune cell infiltration, it may capture biological signals that transcend organ-specific staging systems. This cross-cancer perspective is particularly valuable for stage I tumors, where conventional TNM staging frequently fails to identify high-risk patients who might benefit from closer surveillance or adjuvant therapy. Although prospective validation is required, the cost-effectiveness and scalability of this image-based approach position it as a practical bridge between advanced artificial intelligence and everyday clinical decision-making. Ultimately, such tools could reduce disparities in cancer care by enabling accurate prognostic assessment without the prerequisite of expensive molecular profiling.

However, this study has several important limitations. First, the external validation cohort included only 9 of the 24 cancer types due to data availability. Therefore, generalizability to other cancer types (e.g., ACC, CHOL, and ESCA) requires further validation. Second, the drop in HR from 4.823 to 2.092 suggests overfitting or cohort heterogeneity, indicating that additional external datasets are needed. Third, the variable follow-up times across cohorts might affect DFS event capture, and the absence of multi-omics data (genomic and transcriptomic) in the validation cohort limits integrative analyses. Finally, inherent retrospective biases should be addressed in future prospective studies. While we performed color normalization, batch effects from different scanners and staining protocols may persist, and inherent retrospective biases should be addressed in future prospective studies. We are currently planning prospective validations and expanding the patient cohort to include more cancer types and diverse ethnic populations.

## 5. Conclusions

Our deep learning-based multi-cancer prediction analysis for disease-free survival has shown promising prognostic improvements across multiple cancer types. However, given the moderate performance in external validation and the need for broader validation across all 24 cancer types, this multimodal prediction system should be considered a proof-of-concept tool rather than a mature clinical instrument. The multimodal prediction system could become a practical and valuable tool for diverse cancer management after further technical refinements, multicenter validations, and prospective studies.

## Figures and Tables

**Table 1:** Basic clinical characteristics of patients from three independent cohorts.

	TCGA Dataset (7392)	CPTAC Dataset (545)	General Dataset (919)
**Age (years)**	59.9 ± 13.8	61.0 ± 12.0	64.2 ± 11.0
**Sex**			
Male	3605 (48.8%)	129 (23.7%)	763 (83.0%)
Female	3787 (51.2%)	211 (38.7%)	143 (15.6%)
Unknown	0	205 (37.6%)	13 (1.4%)
**Stage**			
I	1874 (25.4%)	65 (11.9%)	443 (48.2%)
II	1917 (25.9%)	141 (25.9%)	84 (9.1%)
III	1470 (19.9%)	114 (20.9%)	37 (4.1%)
IV	720 (9.7%)	21 (3.9%)	22 (2.4%)
Unknown	1411 (19.1%)	204 (37.4%)	333 (36.2%)
**Status**			
Dead or with tumor	1407 (19.0%)	52 (9.5%)	106 (11.5%)
Alive or without tumor	5985 (81.0%)	493 (90.5%)	813 (88.5%)
**Follow-up time**	36.0 ± 35.4	16.1 ± 9.8	55.1 ± 21.1
**Tumor type**			
ACC	53 (0.7%)		
BLCA	370 (5.1%)		261 (28.4%)
BRCA	1013 (13.7%)	94 (17.2%)	
CESC	246 (3.3%)		
CHOL	35 (0.5%)		
COAD	395 (5.3%)	130 (23.9%)	
ESCA	153 (2.1%)		
HNSC	424 (5.7%)		
KICH	65 (0.9%)		43 (4.7%)
KIRC	479 (6.5%)	101 (18.5%)	262 (28.5%)
KIRP	258 (3.5%)		33 (3.6%)
LIHC	347 (4.7%)		
LUAD	424 (5.7%)	128 (23.5%)	
LUSC	409 (5.5%)		
MESO	56 (0.8%)		
OV	91 (1.2%)		
PAAD	174 (2.4%)		
PRAD	396 (5.4%)		320 (34.8%)
READ	136 (1.8%)		
SKCM	388 (5.2%)		
STAD	367 (5.0%)		
TGCT	132 (1.8%)		
THCA	491 (6.6%)		
UCEC	490 (6.6%)	92 (16.9%)	

**Table 2:** MC-DFS performance in each cancer type of the training and verification cohorts.

	Training Cohort			Verification Cohort		
Tumor Type	AUC	95% CI	*p*	AUC	95% CI	*p*
ACC	0.735	0.596–0.847	0.001			
BLCA	0.625	0.574–0.675	<0.0001	0.644	0.583–0.702	0.0007
BRCA	0.739	0.710–0.765	<0.0001	0.636	0.530–0.733	0.0152
CESC	0.727	0.666–0.781	<0.0001			
CHOL	0.513	0.339–0.685	0.8972			
COAD	0.686	0.638–0.732	<0.0001	0.717	0.627–0.788	0.0007
ESCA	0.760	0.684–0.825	<0.0001			
HNSC	0.629	0.582–0.676	<0.0001			
KICH	0.611	0.482–0.729	0.3809	/	/	/
KIRC	0.738	0.696–0.777	<0.0001	0.705	0.655–0.751	<0.0001
KIRP	0.836	0.785–0.879	<0.0001	0.709	0.525–0.853	0.0318
LIHC	0.672	0.620–0.721	<0.0001			
LUAD	0.610	0.561–0.656	0.0005	0.676	0.588–0.756	0.0417
LUSC	0.634	0.568–0.665	0.0006			
MESO	0.662	0.523–0.783	0.034			
OV	0.664	0.558–0.760	0.0038			
PAAD	0.651	0.575–0.722	0.0003			
PRAD	0.793	0.750–0.832	0.0044	0.711	0.661–0.758	<0.0001
READ	0.592	0.505–0.675	0.2999			
SKCM	0.591	0.541–0.641	0.0016			
STAD	0.664	0.613–0.712	<0.0001			
TGCT	0.677	0.590–0.756	0.121			
THCA	0.697	0.654–0.738	0.1559			
UCEC	0.749	0.709–0.787	<0.0001	0.696	0.591–0.787	0.0634

**Table 3:** MC-DFS results, demonstrating that it could act as an independent risk factor in most cancer types.

	Training Cohort			Verification Cohort		
Tumor Type	Cox	95% CI	*p*	Cox	95% CI	*p*
ACC	106.4366	2.2096–5127.0396	0.0188			
BLCA	8.2939	2.6773–25.6932	0.0003	24.6786	3.6892–165.0868	0.001
BRCA	62.1681	20.5139–188.4022	<0.0001	0.0098	0.0000–3710.0072	0.4826
CESC	158.8209	23.2509–1084.8642	<0.0001			
CHOL	4.7757	0.1200–190.1283	0.4079			
COAD	15.6218	3.2678–74.6794	0.0006	440.9047	6.7254–28,904.7831	0.0045
ESCA	34.0913	5.6587–205.3877	0.0001			
HNSC	4.9696	1.9839–12.4489	0.0007			
KICH	35.0011	0.9433–1298.6963	0.055	NA		NA
KIRC	48.7394	16.8515–140.9686	<0.0001	97.2263	10.7561–878.8476	0.0001
KIRP	374.2544	38.8494–3605.3673	<0.0001	15,096.86	3.7114–61,409,092	0.024
LIHC	23.7495	5.0270–112.2026	<0.0001			
LUAD	5.4981	1.8580–16.2700	0.0022	101.3655	2.6105–3936.0052	0.0138
LUSC	7.5489	2.0933–27.2231	0.0021			
MESO	16.0146	2.7653–92.7436	0.0021			
OV	4.6551	0.9538–22.7197	0.0585			
PAAD	7.1367	1.8090–28.1543	0.0052			
PRAD	162.9643	2.1395–12,413.0546	0.0219	98.6127	20.2497–480.2273	<0.0001
READ	9.8674	0.1912–509.1110	0.2576			
SKCM	4.9771	1.8203–13.6081	0.0019			
STAD	14.6264	4.7873–44.6874	<0.0001			
TGCT	13.7078	0.0086–21,847.6412	0.4887			
THCA	1316.7505	24.6483–70,342.7866	0.0004			
UCEC	72.9883	17.4444–305.3871	<0.0001	65.2686	0.5789–7358.4703	0.0846

## Data Availability

Primary data are available from Atlas (https://portal.gdc.cancer.gov/), accessed on 1 July 2024, and the Clinical Proteomic Tumor Analysis Consortium (https://www.cancerimagingarchive.net/), accessed on 1 July 2024; these databases are openly accessible for research purposes without specific permission. Other private data can only be reasonably requested from the corresponding authors. Clustering-constrained attention multiple instance learning method code is available on GitHub at https://github.com/mahmoodlab/clam (accessed on 1 July 2024).
